# Parasites modulate the gut-microbiome in insects: A proof-of-concept study

**DOI:** 10.1371/journal.pone.0227561

**Published:** 2020-01-14

**Authors:** Brian L. Fredensborg, Inga Fossdal í Kálvalíð, Thor B. Johannesen, C. Rune Stensvold, Henrik V. Nielsen, Christian M. O. Kapel

**Affiliations:** 1 Section for Organismal Biology, Department of Plant and Environmental Sciences, University of Copenhagen, Frederiksberg, Denmark; 2 Department of Microbiology and Infection Control, Statens Serum Institut, Copenhagen, Denmark; University of Lincoln, UNITED KINGDOM

## Abstract

Host-parasite interactions may be modulated by host- or parasite-associated microbes, but the role of these are often overlooked. Particularly for parasites with intestinal stages (either larval or adult), the host gut microbiome may play a key role for parasite establishment; moreover, the microbiome may change in response to invading parasites. Hypothesis testing at the organismal level may be hampered, particularly in mammalian definitive hosts, by ethical, logistical, and economical restrictions. Thus, invertebrates naturally serving as intermediate hosts to parasites with complex life cycles may inform the development of mammalian models as an early-stage host-parasite model. In addition, several important pathogens are vectored by insects, and insect gut microbiome-pathogen interactions may provide essential base-line knowledge, which may be used to control vectorborne pathogens. Here, we used the grain beetle, *Tenebrio molitor*, a host of the tapeworm *Hymenolepis diminuta*, to explore interactions between infection status and resident gut microbiota at two pre-determined time points (day two and seven) post infection. Using 16S/18S microbial profiling, we measured key parameters of the composition, relative abundance, and diversity of the host gut bacteriome and mycobiome. In addition, we quantified the systemic beetle immune response to infection by Phenoloxidase activity and hemocyte abundance. We found significant changes in the gut bacteriome and mycobiome in relation to infection status and beetle age. Thus, the relative abundance of Proteobacteria was significantly higher in the gut of infected beetles and driven mostly by an increased abundance of Acinetobacter. In addition, the mycobiome was less abundant in infected beetles but maintained higher Shannon diversity in infected compared with non-infected beetles. Beetles treated with a broad-spectrum antibiotic (Tetracycline) exhibited significantly reduced parasite establishment compared with the untreated control group, indicating that the host microbiome may greatly influence hatching of eggs and subsequent establishment of *H*. *diminuta* larvae. Our results suggest that experimental work using invertebrates may provide a platform for explorative studies of host-parasite-microbe interactions and their underlying mechanisms.

## Introduction

There is increasing evidence that host-parasite interactions are influenced by host- and/or parasite associated microbes [1–2]. Priority has been given to the increasingly recognized role of the host gut microbiome to organism health, suggesting that the microbiome contributes to the maintenance of gut homeostasis, local immune function, and presumably also systemic immune responses reaching beyond the gut environment [3–5]. In humans, accumulating evidence link the gut microbiome to both dietary preference [6–7] and the occurrence of several chronic diseases, e.g., inflammatory bowel diseases [8–10], type 2 diabetes [11], and atherosclerotic cardiovascular disease [12].

Helminths and protozoa residing in the gut may alter the composition of the gut microbiome [13–19], and those interactions may have important downstream effects on host immunity and gut homeostasis [2,20–23]. Little, however, is known about larval stages of helminths that are transmitted faecal-orally, the presence of which in the gut is transitory because they penetrate the gut epithelium and migrate to other host tissues. Larvae might be able to stimulate alterations in gut microbial composition by providing resources for bacterial growth from chitinous-rich egg shells, change the physical environment of the gut, and/or induce local inflammatory responses from breaching the gut epithelium as part of their migration trajectory.

The gut microbiome may also have important direct and indirect effects on parasite establishment, indicating a three-way interaction between host, gut microbes, and the parasite [24–29]. However, parasite-gut microbiome interactions still represent a relatively recent field of study, and the underlying mechanisms of parasite-induced gut microbiome alterations and host immune modulation remain an undeveloped field of research [2].

The use of mammalian animal models to study host-parasite interactions has a long history and accounts for most of our knowledge on e.g. immunomodulatory effects of helminths on their hosts [30], which has informed the treatment of autoimmune diseases in humans [31]. However, there are important ethical and logistical restrictions associated with the use of mammals in experimental research, and sample sizes of vertebrates are restricted by animal ethics legislation according to the 3R’s (reduce, refine, replace [32]), and husbandry and maintenance costs. Thus, alternative animal models, which allow fast hypothesis testing at both tissue and organismal level, may both reduce and refine subsequent testing that involves conventional animal models.

Insects serving as intermediate hosts of larval stages of helminths or as hosts to other pathogens potentially provide suitable models to address basic conceptual ideas and hypotheses on host-parasite interactions [33–34]. Insects also serve as vectors to pathogens residing in the gut where the gut microbiota may influence the biology of the pathogen and its capacity to transmit to a new host [27–29]. There is therefore an increasing interest in utilizing insect vector microbial composition to control vector-borne diseases [35–36].

The use of insects in research has a long history within genetic, behavioral and physiological sciences. Insects have been used to elucidate important immunological pathways (the Toll pathway was first described in *Drosophila melanogaster* [37]), and to study interactions between bacterial pathogens and the innate immune system, which in insects is similar to that observed in higher vertebrates including humans [33,38]. In addition, insects may also provide basic information on gut-microbiota interactions in the absence of infection because of the relative simplicity of microbial diversity compared with that of vertebrates [39–44].

The present study used the grain beetle *Tenebrio molitor*, which is a natural intermediate host of the tapeworm *Hymenolepis diminuta*, to explore basic interactions between a migratory larval helminth and the gut microbiota of its host. Adult *H*. *diminuta* reside in the small intestine of rats releasing egg-filled proglottids into the feces and thereby further into the external environment. *Tenebrio molitor* and other omnivorous beetles ingesting infected rat feces serve as intermediate hosts. Following ingestion, *H*. *diminuta* eggs hatch into active oncosphere larvae by mechanical rupture by the mandibles and subsequent enzymatic activation in the gut of the beetle [45]. Oncospheres penetrate the intestinal epithelium within 120 minutes of ingestion by the action of hooks and proteolytic enzymes, and migrate to the body cavity where they encyst into infective cysticercoids within 14 days at 25 ºC [46].

The *T*. *molitor*-*H*. *diminuta* association is a well-known model for studies on host-parasite ecology [34], and experimental infection protocols for standardizing parasite infection age and inoculation load exist [47]. In addition, the model is suitable for testing the effects of potential anthelmintics on adult parasites [48–49]. *Hymenolepis diminuta* is known to modify the gut microbiome in the rat definitive host [14–15], and recent proteomics studies indicated several proteomic similarities between the adult and larval stages of *H*. *diminuta* [50–51]. We therefore hypothesize that a grain beetle model may offer an appropriate alternative to a rat model for testing the inverse association between the host gut microbiome and helminth infection. Hence, the beetle model may be used to identify whether the transitory nature of the oncosphere-gut interactions is sufficient to produce persistent alterations in the host gut microbiome.

Here, we used 16S/18S rDNA sequencing of the gut microbiome of *T*. *molitor* to identify changes in the composition of bacteria and fungi upon experimental exposure to *H*. *diminuta* eggs. In addition, we quantified the effect of parasite infection on the generalized constitutive immune response of the beetles to explore any correlation to the composition of the gut microbiome. In a second experiment, we used artificially induced gut perturbation by antibiotics to test the potential effect of the gut microbiota on the establishment of *H*. *diminuta*.

## Methods

### The influence of parasite infection on host gut microbiota

#### Sterilizing *Hymenolepis diminuta* eggs

*Hymenolepis diminuta* eggs were sterilized using the protocol by [52] with modification. *H*. *diminuta* eggs were harvested from 1–7-day-old rat feces from two infected rats (*Rattus norvigecus*, Wistar strain) hosted at the Panum Institute, University of Copenhagen (Permit no. 2014-15-0201-00387). Twenty grams of fecal pellet was collected from cage bedding material and soaked in water for one hour. Subsequently, the fecal suspension was poured through a double layer of gauze (mesh size, 1 x 1 mm) followed by a 100-μL mesh sieve to separate large fragments and debris, and the eggs were then collected on a 63-μL mesh sieve and transferred to a 50-mL Falcon tube. Following sedimentation, the supernatant was removed and replaced with 30 mL of flotation fluid consisting of saturated salt solution and centrifuged at 100 x *g* for 2 min. Subsequently, 10–15 mL of the supernatant containing the eggs was collected in a new Falcon tube, washed using MilliQ water, and centrifuged 4–5 times as described above. Following sedimentation, the supernatant was removed, and 10 mL of 0.1% Rodalon was thoroughly mixed with the sample, left for 20 min, and centrifuged and washed (using MilliQ water) 4–5 times. The concentration of eggs in 10 μL was calculated. Twenty microliters of the suspension was plated on Luria-Bertani (LB) agar plates and incubated at 37 ºC for 48 h. Plates were inspected for bacterial growth after 24 h and 48 h. After the sterilization process, the viability of the eggs was checked *in vitro* according to [53]. Briefly, a droplet of egg suspension was transferred to a microscope slide, a cover slip was added, and slight pressure was applied to the cover slip to provide mechanical pressure and rupture of the egg capsule, mimicking the action of beetle mandibles during ingestion of *H*. *diminuta* eggs, and activating the infective oncosphere larvae as observed by hook movement.

#### Experimental infection

**Fig 1:**
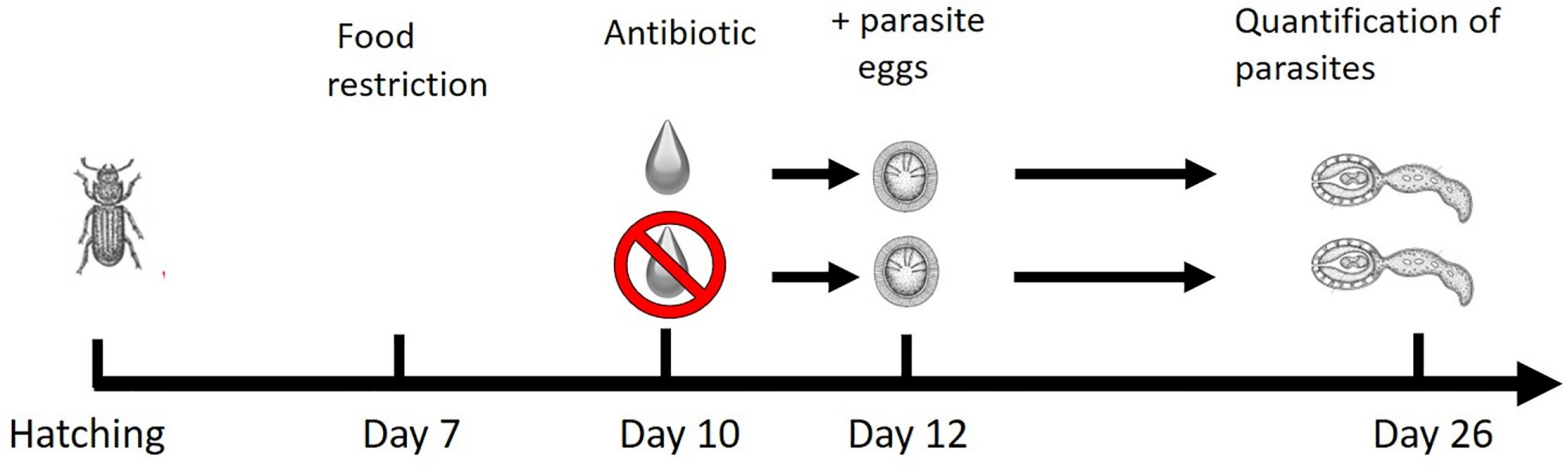
Diagram of experimental design. Experimental design of the effect of antibiotic treatment on parasite establishment (number of cysticercoids in body cavity). See text for details.

More than 100 5–7-day-old beetles were starved for three days and then exposed to sterilized *Hymenolepis diminuta* eggs (see above) or water (control)(Fig 1). Beetles were transferred individually to petri dishes (diameter of 55 mm) lined with Whatman TM filter paper, and a cover slip (15x15 mm) was placed in the center of the dish. Each beetle was provided with 5 μL of a suspension of sterilized *H*. *diminuta* eggs, containing approximately 300 eggs mixed with 0.02 g of autoclaved apple and prune gravy (Hipp.dk product number 44946) placed on the cover slip. Control beetles received the autoclaved apple and prune gravy mixed with 5 μL of MilliQ water. The beetles were kept individually in darkness for 24 hours to provide them with an opportunity to eat the gravy. After 24 hours, the petri dishes were inspected, and the beetles that had not ingested the entire droplet of gravy were excluded from the experiment. From the remaining beetles, 32 experimentally infected individuals and 32 control beetles were transferred to each a plastic container (185 x 122 x 72.1 mm) supplied with *ad libitum* organic whole grain oat flakes and slices of fresh potato.

### Beetle immune parameters

On days two and seven post infection (PI), 16 uninfected and 16 infected beetles were haphazardly selected from the stock population. Hemolymph (2 μL) was sampled from each beetle by puncturing the neck-region between the head and the pronotum with a needle and collecting a droplet of hemolymph with a pipette. The collected hemolymph was transferred immediately to PCR tubes containing 10 μL of 10 x 1 PBS (Phosphate buffered saline, Amresco, code 0780-1PK) kept on dry ice. Following hemolymph collection, the beetles were euthanized by swiftly separating the head from the body by the use of forceps, dissected, and for each beetle, the gut was removed and stored in 70% Ethanol until extraction of DNA from gut contents (see below). Hemolymph from four beetles was pooled together and used for a phenoloxidase (PO) assay. The PO assay was performed in duplicates, each containing 30 μL of the pooled hemolymph according to Dhakal et al. 2018. Samples were loaded on a plate reader (SpectraMax® i3, Molecular Devices), and readings were obtained using the program Soft Max pro 6.4. for a single reading at a wavelength of 595 nm. PO activity was determined at 490 nm, and readings were collected every two minutes for one h at 28 ºC.

Ten microliters of hemolymph were added to 5 μL 4% Formaldehyde solution and used for hemocyte counting. Hemocytes were counted using a compound microscope at 100X magnification, using 9 μL Fast-Read 102® Counting slides (Immune systems Ltd, BVS100, Lot: 910512).

Extra beetles from the experimental infection group were kept alive for 14 days, to verify infection status and to count the number of cysticercoids in the hemocoel by dissection to verify parasite establishment success.

### Mini DNA-extraction with CTAB II

The beetle gut was stored in 1.5 mL Eppendorf tubes with 70% ethanol. Prior to processing, the ethanol in each sample was removed. Then, the beetle gut was mascerated, and 500 μL CTAB-buffer was added to each sample together with 1 μL 2- mercaptoethanol. The samples were then heated to 60 ºC for 1 h and shaken at regular intervals. Thereafter, 500 μL of chloroform/isoamyl alcohol (24:1) were added, and the samples were stirred and centrifuged for 10 min at 11,000 rpm. A total of 350 μL (2 x 175 μL) of the supernatant were transferred to a new Eppendorf tube, and the remaining liquid was discarded. A total of 210 μL of 2-propanol were added to and mixed with each sample, for overnight incubation at -20 ºC. The following day, DNA was centrifuged at 11,000 rpm for 10 min at 4 ºC. The supernatant was discarded, and 500 μL of 70% ethanol were added before the samples were again centrifuged for 10 min at 11,000 rpm at 4 ºC. The supernatant was removed, and the DNA washed with 70% ethanol a few times, air-dried, and then 50 μL of 1 x TE-buffer was added. For each batch of DNA extraction, a “negative” control was included containing only buffers.

Following DNA extraction of the beetle gut, samples were further processed at the Department of Bacteria, Parasites and Fungi, Statens Serum Institut (SSI), Copenhagen, Denmark for total nuclear ribosomal gene amplification and sequencing (see below).

#### Primer design for ribosomal gene amplification and sequencing

The 16S/18S assay was similar to the one previously used [54–55]. Briefly, the 16S rRNA gene was targeted for amplification, using a modified version of the published universal prokaryotic primers 341F/806R, targeting the V3-V4 hyper-variable regions (pmid 15696537). The forward primer had three additional nucleotides attached to the 5’ end (ACTCCTAYGGGRBGCASCAG, 341F3) and the reverse primer had five additional nucleotides attached to the 5’ end (AGCGTGGACTACNNGGGTATCTAAT, 806R5).

The 18S rRNA gene was selected as target gene to ensure the broadest possible spectrum of eukaryotic species (parasites and fungi) to be amplified by as few primer sets as possible, when assuming that nuclear ribosomal genes (18S) would be the most inter-species conserved gene. Three different primer sets were chosen, G3F1/G3R1 (GCCAGCAGCCGCGGTAATTC / ACATTCTTGGCAAATGCTTTCGCAG), G4F3/G4R3 (CAGCCGCGGTAATTCCAGCTC / GGTGGTGCCCTTCCGTCAAT) and G6F1/G6R1 (TGGAGGGCAAGTCTGGTGCC / ACGGTATCTGATCGTCTTCGATCCC). G3 and G6 primers target the hyper-variable regions V3-V4 of the 18S rDNA gene, and G4 primers target V3-V5.

#### Library preparation

Purified genomic DNA from each sample was initially amplified using the 16S and 18S primers. The 16S and 18S rDNA was amplified in a 25μL volume, using the REDExtract-N-Amp PCR ReadyMix (Sigma-Aldrich, St Louis, MO, USA) with 0.4 μM of each primer and 2 μL of template. The 16S PCR was run with an initial denaturation at 95°C for 2 min, 20 cycles of 95°C for 30 sec, 60°C for 1 min, and 72°C for 30 sec, and a final elongation at 72°C for 7 min. The 18S PCR setup was run with an initial denaturation at 95°C for 3 min, 20 cycles of 95°C for 1 min, 60°C for 1 min, and 72°C for 30 sec, and a final elongation at 72°C for 4 min. These PCR runs are referred to as PCR1 or amplification PCR. The products from PCR1 were prepared for sequencing by a second PCR (PCR2 or adaptor PCR), using the same PCR protocol as described above. PCR2 attached an adaptor A, an index i5, and a forward sequencing primer site (FSP) to the 5’ end of the amplicons and an adaptor B, an index i7, and a reverse sequencing primer site (RSP) to the 3’ end of the amplicons. DNA was quantified using the Quant-ITTM dsDNA High Sensitive Assay Kit (Thermo Fisher Scientific), and PCR2 products were pooled in equimolar amounts across samples. Undesirable DNA amplicons were removed from the pooled amplicon library (PAL) by Agencourt AMPure XP bead (Beckman Coulter) purification by a two-step process as follows: Firstly, DNA fragments below a length of 300 nt were removed by a PAL AMPure beads 10:24 ratio, following the manufactures protocol and eluted in 40 μL TE buffer (AM1). Secondly, large DNA fragments with lengths above 1 kbp were removed by an AM1 to AMPure beads 10:16 ratio. The resulting AMPure beads-purified PAL (bPAL) was diluted to its final concentration of 11.5 pM DNA in 0.001 N NaOH and used for sequencing on the Illumina MiSeq desktop sequencer (Illumina Inc., San Diego, CA 29122, USA). The library was sequenced with the 500-cycle MiSeq Reagent Kit V2 in a 2 x 250nt setup (Illumina Inc., San Diego, CA 29122, USA).

### The influence of the beetle gut microbiota on the establishment success of *Hymenolepis diminuta*

A total of 150 grain beetles, five to seven days post hatching, were starved for three days and transferred individually to petri dishes (diameter of 55 mm lined with WhatmanTM filter paper), and provided with 10 μL of antibiotics (tetracycline hydrochloride, CAS nr. 64-75-5, 10 mg/mL) mixed with 10 μL of autoclaved apple/elderflower juice (Løgismose, unfiltered) on a 15 x 15 mm cover slip. The choice of antibiotic agent was based on a pilot study where study beetles were offered two different types of antibiotics in two concentrations (Pen Strep (5,000 U/mL penicillin and 5,000 μg/mL streptomycin or 10,000 U/mL penicillin and 10,000 μg/mL streptomycin) or tetracycline (5 mg/mL or 10 mg/mL), and the control beetles received water. The gut was extracted from each beetle (upon euthanization) after 48 hours and stored in 70% ethanol until DNA extraction and 16S/18S rDNA sequencing (see above). Since beetles treated with tetracycline exhibited the most prominent shifts in gut microbiota compared to controls, this agent was used in the present study (S1 Fig).

Beetles were kept in darkness for 1–2 hours to allow them enough time to drink the droplet containing the antibiotic. The beetles that did not consume the entire droplet were excluded from the experiment. After 48 hours, the beetles were exposed to approximately 300 sterilized *H*. *diminuta* eggs (see above) in 0.02 grams of autoclaved apple and prune gravy (Hipp.dk, product no. 44946) on a 15 x 15 mm cover slip and kept in darkness for 24 hours. Control beetles were exposed to the same treatment, but they received 10 μL of sterilized water instead of antibiotics.

The beetles that received antibiotics and *H*. *diminuta* infection were kept under sterile conditions (i.e., the box was washed and rinsed with 70% Ethanol) for 14 days after infection and then dissected to quantify the number of cysticercoids present in the body cavity, and the gut was removed and kept in 1.5 mL Eppendorf tubes with approximately 1 mL 70% Ethanol until DNA extraction. The beetles received access to autoclaved oat flakes *ad libitum*, and autoclaved water from a 1.5 mL vial plugged with autoclaved cotton wool. Oats, water and cotton were replaced every third day and the box was rinsed with 70% Ethanol. The box containing the beetles was kept inside another bigger box and stored on a sterile bench until the day of dissection.

### Statistics and data analysis

Raw reads were analyzed and classified taxonomically using the “BION-meta” package, which performed quality-trimming, read pairing, and chimera filtering before performing taxonomic classification of sequences. Classification of 16s rRNA sequences was based on The Ribosomal Database Project (RDP) database while sequences from the three 18s rRNA targets were classified against the SILVA database.

All analyses of classified sequence data were conducted using the statistical software package R v. 3.2.3 1. Microbiome data was handled using the add-on package ‘phyloseq’ v. 1.16.2 2 and visualized with ‘ggplot2’ v. 2.1.0 3 and plotly. Analysis of bacterial microbiome was based on data rarefied to 26040 sequences (the lowest number of sequences found in any sample) and fungal data was rarefied to 817 sequences leading to the exclusion of two samples from further analysis of fungal composition.

Differences between treatment groups were assessed with barplots and Principal Coordinates Analysis (PCoA) plots using Bray-Curtis dissimilarities and tested with Analysis of Similarity (ANOSIM). Distributions of observed number of species and Shannon index between sample groups were visualized with boxplots and tested using Kruskal-Wallis tests (when testing more than two groups) or Mann-Whintey rank sum tests (when testing two groups). Differences in relative abundance of individual genera between groups were tested with pairwise Mann-Whitney tests. Multiple testing correction was performed based on False discovery rate (FDR) and a p-value below 0.05 was considered statistically significant.

Barplots of most abundant genera were created by agglomerating counts within each genus. Counts were normalized to percentages per sample and the ten species with the highest sum of percentage were kept.

### Ethics approval

The rats used to supply infection material for the beetles were house under experimental license from the Danish Department of Justice (License no. 2014-15-0201-00387– section C1). No ethics approval is necessary for work with insects.

## Results

### The influence of *Hymenolepis diminuta* on beetle gut microbiota

**Fig 2:**
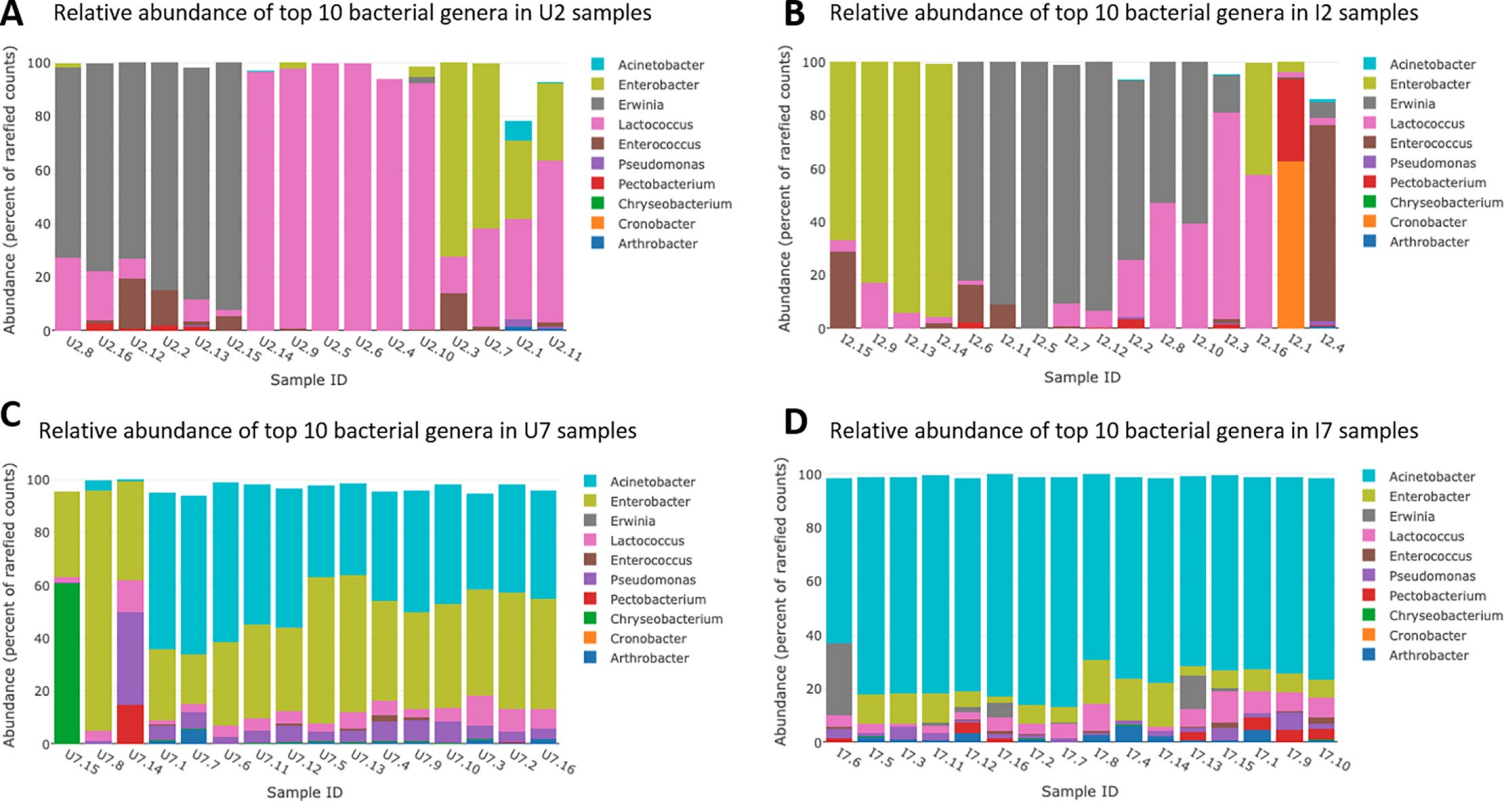
Relative abundance of the bacteriome in the beetle gut in relation to time and infection status. Relative abundance of the ten most abundant prokaryotic genera in the intestinal tract of the grain beetle, *Tenebrio molitor* in uninfected (A and C) and experimentally *Hymenolepis diminuta* -infected beetles (B and D) on day two (A and B) and seven (C and D) post infection.

Operational Taxonomical Units (OTU) were assigned to seven bacterial phyla (Proteobacteria, Bacteroides, Firmicutes, Actinobacteria, Spirochaetes, Fusobacteria, and Deinococcus-Thermus, OTU = 252), and four fungal phyla (Ascomycota, Basidiomycota, Entomophtoromycota, and Mucoromycotina, OTU = 114). The most abundant bacterial phylum was Proteobacteria accounting for 72.2% and 91.9% in infected beetles on day two and seven, respectively, and 45.0% and 87.1% in non-infected beetles on day two and seven, respectively. The most abundant genera on day two were *Erwinia* and *Lactococcus*, while *Acinetobacter* and *Enterobacter* were the most abundant genera on day seven post infection for both infected and non-infected beetles (Fig 2).

**Fig 3:**
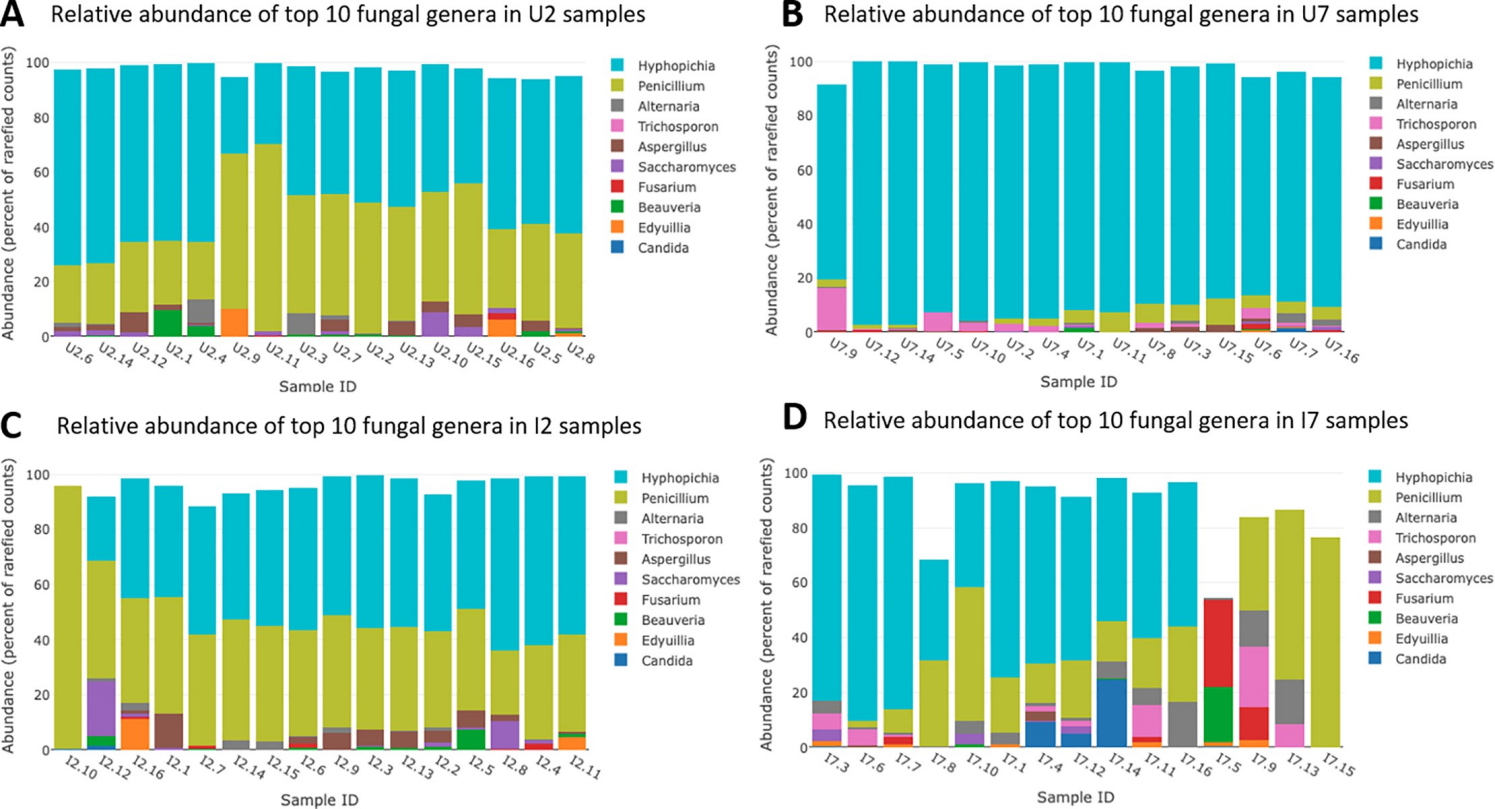
Relative abundance of the mycome in the beetle gut in relation to time and infection status. Relative abundance of the ten most abundant eukaryotic genera in the intestinal tract of the grain beetle, *Tenebrio molitor* in uninfected (A and C) and experimentally *Hymenolepis diminuta* -infected beetles (B and D) on day two (A and B) and seven (C and D) post infection.

We observed significant effects of both beetle infection status and time on the relative abundance of several bacterial and fungal genera (Figs 2 and 3) (ANOSIM, R = 0.50, p = 0.001).

On day seven, a significant difference was found in the abundance of 12 genera of bacteria and three genera of fungi, representing five phyla (three bacterial and two fungal). Of these, five bacterial genera were enriched in infected beetles, while seven were depleted. Likewise, one fungal genus was enriched, while two were depleted in infected beetles. Numerically, the greatest differences in the relative abundance of the bacteriome between uninfected and infected beetles included a reduction of *Enterobacter* (-60%, p<0.001) and a concurrent increase in the relative abundance of *Acinetobacter* in infected beetles (1315%, p<0.001) (Fig 2). In the mycobiome, a reduction of the dominant genus *Hypopichia* was observed in infected beetles (-82%, p = 0.019) (Fig 3).

**Fig 4:**
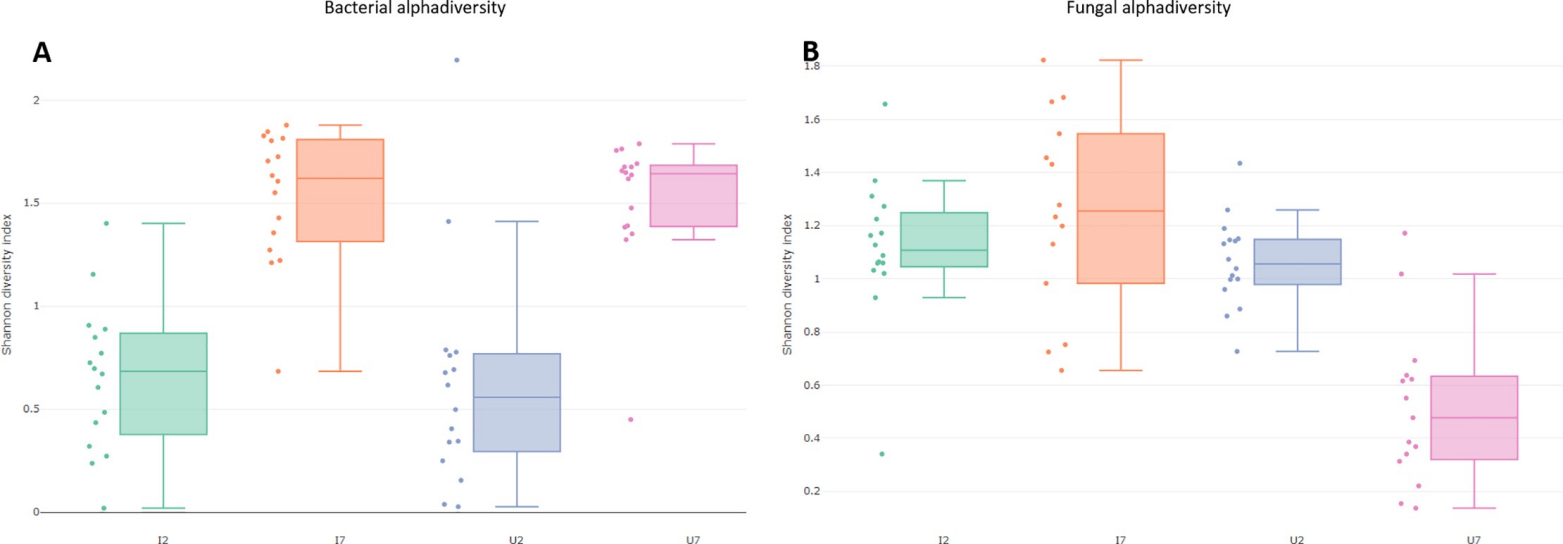
Shannon diversity in relation to time and infection status. Alpha diversity of prokaryotes (A) and eukaryotes (B) in the intestinal tract of *Tenebrio molitor* in relation to infection status with *Hymenolepis diminuta* and time (day two or seven days post infection). The Shannon index for the mycobiome (B) was significantly reduced in the uninfected beetles on day seven in comparison to the infected group (Mann Whitney test, P<0.001).

**Fig 5:**
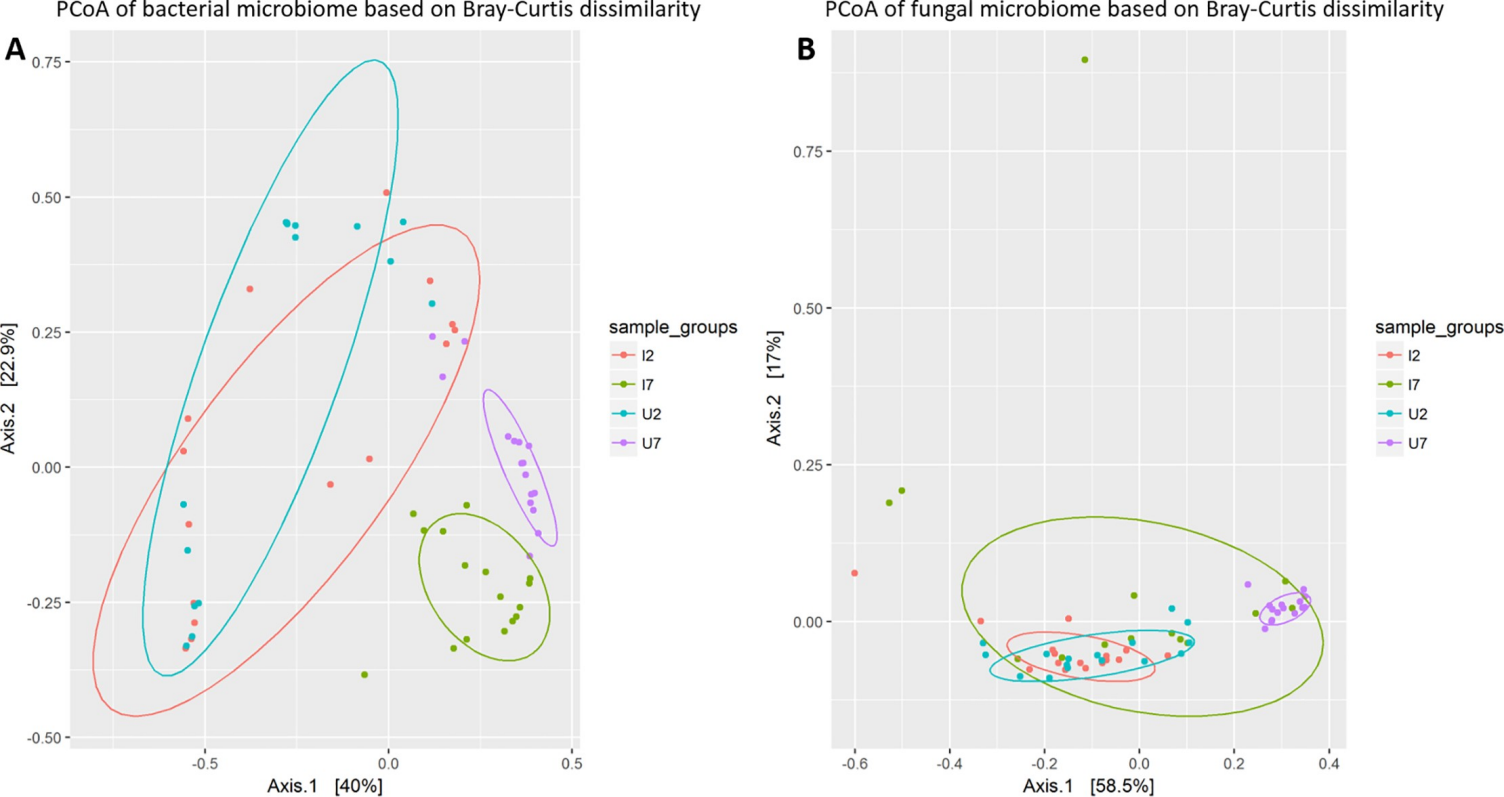
Microbial and fungal composition in beetle gut in relation to time and infection status. Principal Coordinates Analysis (PCoA) plots based on Bray-Curtis dissimilarity on the composition of bacteria (A) and fungi (B) in the intestinal tract of *Tenebrio molitor* according to *Hymenolepis diminuta* infection status (U = uninfected or I = infected), and time (day two or day seven). Both the bacteriome and the mycobiome composition differed significantly between uninfected and *H*. *diminuta*-infected beetles on day seven p.i. (ANOSIM, P = 0.001).

The alpha diversity of bacteria (richness and Shannon Index) increased significantly from day two to day seven (p<0.001) with no difference between infected and uninfected beetles on any of the two sampling days (Fig 4A). However, the Shannon Index for the mycobiome decreased significantly in uninfected beetles on day seven (p<0.001), a pattern not observed in infected beetles, which maintained the observed diversity from day two post infection (Fig 4B). The composition of the bacteriome differed significantly over time and according to infection status (Fig 5A). Gut bacteriome composition differed significantly between day two and day seven in both infected (ANOSIM, R = 0.63, p = 0.001) and uninfected (ANOSIM, R = 0.69, p = 0.001) beetles. On day two post infection there was no significant difference between non-infected and infected beetles (ANOSIM, R = 0.063, p = 0.083), while on day seven, the composition of the bacteriome differed significantly between non-infected and infected individuals (ANOSIM, R = 0.68, p = 0.001) (Fig 5A). Similar to the bacteriome, the composition of the mycobiome differed significantly between day two and day seven post infection regardless of infection status (ANOSIM, infected: R = 0.21, p = 0.001, uninfected: R = 0.84, p = 0.001). While there was similarity in the composition on day two (ANOSIM, R = -0.001, p = 0.42), non-infected and infected individuals differed significantly on day seven post infection with dissimilarity among beetles within the infected group (ANOSIM, R = 0.37, p = 0.001) (Fig 5B).

### Influence of beetle gut microbiota perturbation on *Hymenolepis diminuta* establishment

Beetles treated with Tetracycline two days prior to experimental infection exhibited a higher relative abundance of Proteobacteria than control beetles (89.2% versus 57.5%), and a lower relative abundance of Firmicutes compared with the control group (9.4% versus 41.5%) (S1 Fig).

**Fig 6:**
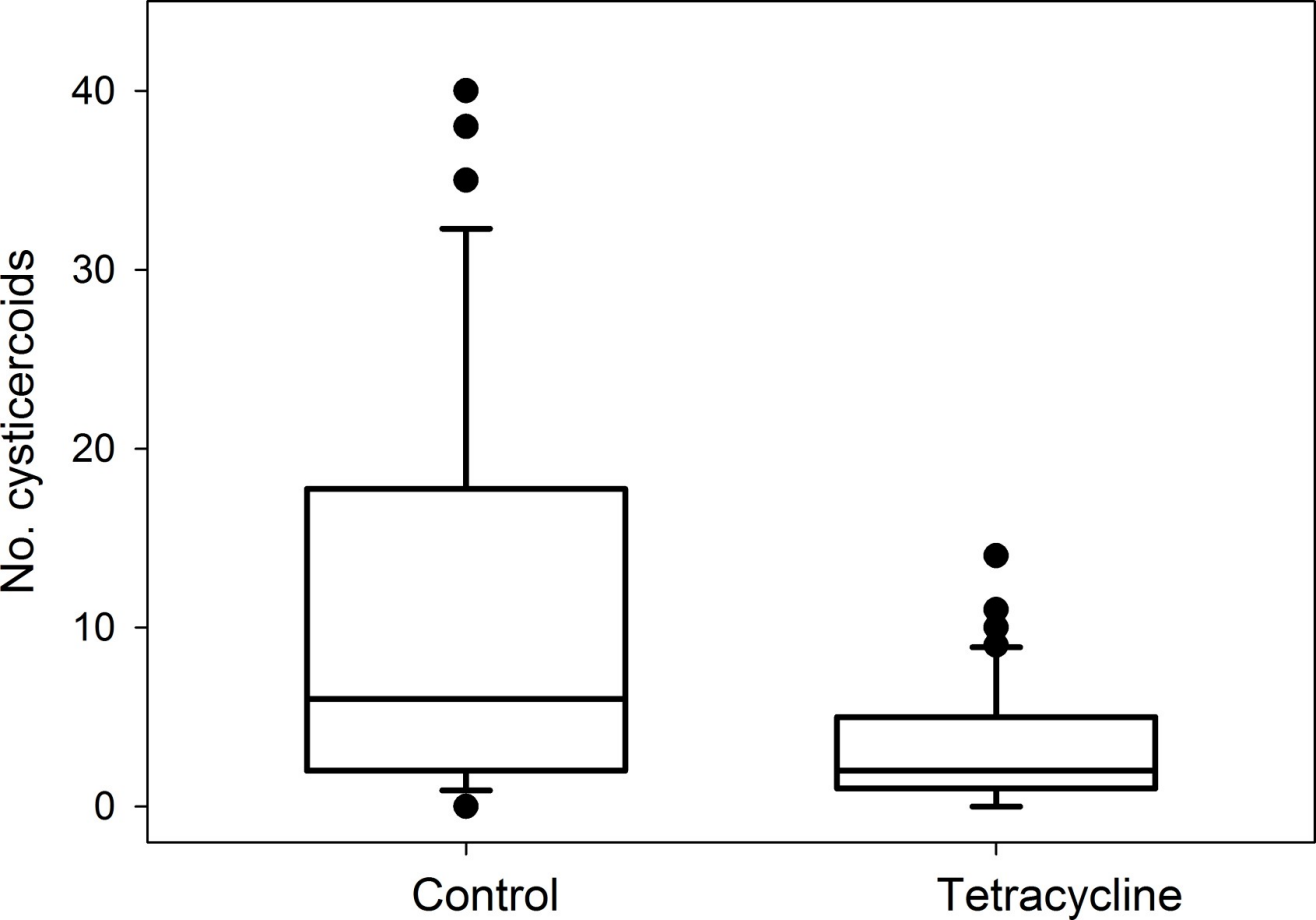
Parasite establishment success in relation to experimental gut dysbiosis. Establishment success of *Hymenolepis diminuta* in *Tenebrio molitor* quantified as the number of cysticercoids established in the beetle hemocoel after exposure to a standardized number of parasite eggs. Sample sizes were n = 38 for control beetles and n = 50 for beetles receiving tetracycline treatment. The box represents the upper, median (black bar), and lower quartiles, respectively. The whiskers represent the 90^th^ and the 10^th^ percentiles, respectively, and outliers are indicated by dots.

Tetracycline-treated beetles displayed a significantly reduced establishment of *H*. *diminuta* cysticercoids compared with the untreated control group (70% reduction, Mann Whitney test, p = 0.0003, Fig 6). The mycobiome was dominated by *Candida* in both treatment groups, but less so in tetracycline-treated beetles compared with control beetles (34% versus 77% of the total abundance).

#### Overall concordance between mycobiome and microbiome

Three overall clusters of the mycobiome were identified based on hierarchical clustering of Bray-Curtis dissimilarities between samples. The fungal clusters were in high concordance with sample time and infection status, with 15/16 I2 and 16/16 U2 samples belonging to cluster 1 and 15/15 U7 samples being grouped into cluster 3 (S2 Fig). Only the I7 group was notably split across all three clusters. Interestingly the I7 group, which was the most diverse in terms of fungal composition, clustered perfectly when grouped by bacteriome composition, mainly due to high relative abundance of *Acinetobacter* in all samples. The majority of U7 samples, which all grouped into fungal cluster 1, also had a unique bacterial composition with high amounts of both *Acinetobacter* and *Enterobacter*.

### Beetle systemic immune response to infection

There was no significant difference in mean PO activity according to beetle age or infection status (Two-way ANOVA: age: F = 0.36, P = 0.56, infection status: F = 1.38, P = 0.26, age x infection status: F = 0.86, P = 0.37). Likewise, there was no significant difference in the abundance of hemocytes in relation to age and infection status (age: F = 2.60, P = 0.13, infection status: F = 0.08, P = 0.78, age x infection status: F = 2.29, P = 0.16).

## Discussion

### Parasite-related effects on host gut microbiota

This study demonstrated that the composition of the beetle gut microbiota (both bacteriome and mycobiome) change significantly within a week after experimental exposure to tapeworm. This is noticeable, as *H*. *diminuta* is only a transient visitor of the host gut, where larval stages penetrate the gut epithelium 30–120 minutes after hatching from the ingested egg [45]. The remaining development of the tapeworm takes place in the hemocoel of the beetle. This may suggest that the continued presence of the parasite in the gut is not an absolute requirement for the changes in the gut microbiome, and further that a continuous stimulation of the gut epithelium by parasite excretory-secretory products or continuous resource competition as described for chronic intestinal helminth infections of vertebrates [15,20] is not an absolute requirement for such changes to take place. Instead, we hypothesize two alternative mechanisms that are not mutually exclusive to explain the observed changes to the beetle gut microbiome.

Firstly, parasite stimulation of the innate immune system of the beetles may have caused the modification of the gut microbiome. The insect immune system consists of a non-specific cellular constitutive component (hemocytes), which is immediately responsive to infectious agents in conjunction with enzyme cascades (phenoloxidase). In addition, the innate immune system contains an inducible localized response by the production and release of specific antimicrobial peptides (AMP), which takes up to 48 hours to take effect but subsequently lasts for weeks [38,56]. These are important in the regulation of potentially harmful bacteria in the gut microbiome and the maintenance of gut homeostasis [57]. Onchosphere larvae penetrate the gut epithelium by the aid of hooks and proteolytic enzymes, a process which potentially stimulates a localized inducible response (AMP) as well as a systemic constitutive response if gut bacteria cross the epithelial barrier and enter the body cavity. Our study did not indicate that gut microbes crossed the gut epithelial barrier since neither hemocyte numbers nor PO activity were upregulated after experimental infection. It is possible that parasite excretory-secretory (ES) products (e.g. exosomes) released immediately after infection may have induced a persistent down-regulation of host gene expression for a constitutive immune response, but at present there is no evidence supporting persistent effects of parasite ES products on host immunity. In contrast, AMP activation may affect the resident microbial composition [58], and our data suggest that the reduction in *Enterobacter* observed for infected beetles on day seven post infection may have facilitated the increased relative abundance of *Acinetobacter* (S3 Fig). The inducible nature of AMP’s could also explain why changes to the composition of the gut microbiome were not detected on day two post infection, but that they were evident on day seven post infection. In future studies, production of antimicrobial peptides or at least their gene expression should be measured in gut epithelial tissue before and several days after experimental infection of the beetles to evaluate the contribution of AMP’s to gut microbiome composition.

Gut penetration by oncospheres may also alter the physical/chemical environment including the redox potential with possible indirect effects on microbial composition. Our results show that the gut of infected beetles by day seven was dominated by the aerobe *Acinetobacter* at the expense of the facultative anaerobe group of *Enterobacter*, and anaerobe members of Firmicutes and Bacteroidetes. Thus, parasite penetration to the much more oxidized environment of the beetle gut cavity could have produced a temporary change in gut redox potential stimulating facultatively aerobe bacteria.

Finally, a previous study proposed that chitin from parasite egg-shells may serve as microbial nutrition, stimulating specific elements of the bacterial flora [59]. In our study, eggs were only introduced once, and we consider it an unlikely cause to the changes in microbial composition observed seven days later.

Whatever the exact mechanism(s) of parasite-induced changes to the host gut microbiome, our study suggests that even transient interactions between larval helminths and the gut microbiome of their hosts may modify the microbial composition of the gut. To our knowledge this has not been documented previously, and it may have general implications for understanding the effect of helminth eggs or larvae (e.g., taeniids and ascarid nematodes), which transiently pass through the gut of intermediate and aberrant hosts (including humans).

Infected beetles displayed a significantly higher alpha diversity of fungi on day seven post infection compared with non-infected individuals (Fig 3B). This difference in diversity reflected a decrease in diversity in the non-infected group in contrast to the stable diversity observed in infected beetles. The difference on day seven was probably due to the reduction in the most dominant genus *Hypopichia* in the infected group, facilitating an increase in a number of other subordinate taxa (e.g. *Penicillum* and *Alternaria*).

There is a high degree of concordance between mycobiome and bacteriome composition among the samples. This is likely caused by treatment and infection status having a unique impact on both, rather than interactions between particular organisms. Both symbiotic and antagonistic relationships between members of the mycobiome and microbiome communities are likely to exist. However, since the effect of treatment and infection are so dominant, and since splitting samples by time and infection status results in very small sample sets, a much larger data set is required to examine interactions between the bacteriome and the mycobiome.

### Effects of host gut microbiota on parasite establishment success

Previous studies on mammalian gut helminths showed that hatching of parasite eggs was facilitated by the resident gut bacteria [24], and that bacterial stimulation of egg hatching was parasite species-specific as close relatives are not equally stimulated by the host gut bacterial composition [60]. In this study, we found a significant reduction in parasite establishment in beetles when the gut microbiota had been perturbed by tetracycline prior to the experimental infection. This suggests that the gut bacteria interact with the eggs of *H*. *diminuta* either directly or indirectly by influencing the abiotic environment facilitating hatching of *H*. *diminuta* eggs. The latter may be very important as hatching of helminth eggs is stimulated by a combination of specific abiotic cues such as gut pH, oxidation-reduction potential, temperature, and enzymatic concentrations [45,61–62]. Given the restricted time of opportunity for hatching and penetration of the gut epithelium (30–120 min)[45], even slight deviations from optimal conditions could greatly reduce parasite establishment success. However, parasite establishment was not completely inhibited as some cysticercoids were recovered also from antibiotic-treated beetles, which is in agreement with observations by [63] who successfully infected germ-free *T*. *molitor* with eggs of *H*. *diminuta*.

Interestingly, the infection of *Plasmodium* in *Anopheles* mosquitos resembles that of *H*. *diminuta* in beetles as *Plasmodium* transiently resides in the mosquito gut before crossing the gut epithelial layer to develop as oocysts in the hemocoel. However, in contrast to *H*. *diminuta*, perturbation of the gut microbiota by antibiotics increases the susceptibility of mosquitos towards *Plasmodium* potentially caused by a substantial increase in microbial abundance following a blood meal in turn eliciting an immune response [64]. In another important vector-borne pathogen, *Trypanosoma cruzi*, the gut microbiome of the triatomine insect vector may also directly interact with the developing parasite through competition for resources [65].

We investigated changes to the host gut microbiome on day two and day seven post infection. However, we did not study if the observed changes were persistent, or if the microbiome composition of the infected group of beetles over time became increasingly similar to that of the non-infected group. This is particularly relevant since the gut involvement of the infection is temporary. Therefore, future studies should sample the host gut microbiome over a longer period (e.g. weeks). Additionally, it would be relevant to examine the effect of host age at the time of infection on gut microbiome composition as a function of infection status. The composition of the gut microbiome clearly changed with host age, but potential interactions between infection status and age are unknown.

## Conclusion

The present study demonstrates that parasites modulate insect host gut microbiomes, and that the composition of the insect gut biome affects parasite establishment success. Hence, insects may be used as an inexpensive and fast model for studying host-parasite-microbiota interactions. Furthermore, this study supports the notion that the insect gut microbiome may contribute important knowledge to future control of vector-borne diseases [35–36]. Due to the different composition of the gut microbiome and the lack of an adaptive immune system of insects, results obtained from our model may not necessarily translate directly to mammalian models. However, an insect model may serve as a generator of generally applicable hypotheses related to host-pathogen-microbe interactions and their underlying mechanisms. For example, our study indicated that helminth larvae migrating through the gut produce changes in the gut microbiota despite the transitory nature of interaction. The insect model has the great advantage of providing complete organism responses to infection, yet remaining cost-effective and ethically sound where large sample sizes and complex experimental designs are warranted. Insects may be relevant in generating new hypotheses related to the mechanistic insights of parasite-gut microbiome interactions, and in directing research questions in more complex e.g. mammalian models. Thus, the insect model provides a way to generate a considerable amount of data, while at the same time reducing the need for vertebrates used in the early stages of research (in agreement with the 3R’s). Insect models may therefore have considerable commercial potential in biomedical research.

## Supporting information

S1 Fig Relative abundance of the ten most abundant A) prokaryotic, and B) eukaryotic genera in relation to treatment at 48 h after treatment with Tetracycline (pooled data from beetles receiving either 5 mg mL^-1^ or 10 mg mL^-1^). Data represent the average relative abundance per treatment (control: n = 5, tetracycline: n = 10).
(TIF)

S2 Fig Barplots of the top 10 genera found in the mycobiome (A) and bacteriome (B) in the 62 samples which had data on both bacterial and fungal composition. Samples are ordered according to a hierarchical clustering of Bray-Curtis dissimilarities between samples, and three overall clusters of the mycobiome have been defined based on this clustering. These three clusters can largely be described as a *Hyphopichia* dominated cluster (cluster 1), a diverse cluster with no *Hyphopichia* at all (cluster 2), and a cluster where *Pennicillium* dominates along with *Hyphopichia* (cluster 3). Tiles below the barplots are color coded to show fungal cluster as well as time (day 2 or 7), and infection status (uninfected = U or infected = I) of samples in order to highlight concordances between bacteriome, mycobiome, and infection status.
(TIF)

S3 Fig Relationship between the abundance (number of reads) of *Enterobacter* and *Acinetobacter* in uninfected beetles (a) and beetles infected with *Hymenolepis diminuta* (b) on day 7 post infection (n = 16 in both). The stipled line represents the best linear fit between the abundance of the two bacterial genera. In uninfected beetles there was a significant negative correlation between the abundance of Enterobacter and Acinetobacter (Spearman Rank Order Correlation, r = -0.64, p = 0.007), while there was no significant relationship between the two bacterial genera in infected beetles (r = 0.027, p = 0.92).
(TIF)

S1 File 16S/18S sequencing data of *Tenebrio molitor* intestines in relation to beetle infection status. (XLSX)

S2 File Sequencing data summary. (XLSX)

S3 File 16S/18S sequencing data of *Tenebrio molitor* intestines in relation to experimental gut dysbiosis from exposure to antibiotics. (XLSX)

S4 File Parasite establishment in *Tenebrio molitor* in relation to exposure to tetracycline. (XLSX)

S5 File Phenoloxidase activity in the hemolymph of *Tenebrio molitor* in relation to infection status day 2 post infection. (XLSX)

S6 File Phenoloxidase activity in the hemolymph of *Tenebrio molitor* in relation to infection status day 7 post infection. (XLSX)

S7 File Hemocyte counts in the hemolymph of *Tenebrio molitor* in relation to infection status. (XLSX)
