# Supplementary figures and images for: Parasites modulate the gut-microbiome in insects: A proof-of-concept study
Source: PLoS One. 2020 Jan 14;15(1):e0227561. doi: 10.1371/journal.pone.0227561 (PMC6959588; doi:10.1371/journal.pone.0227561)

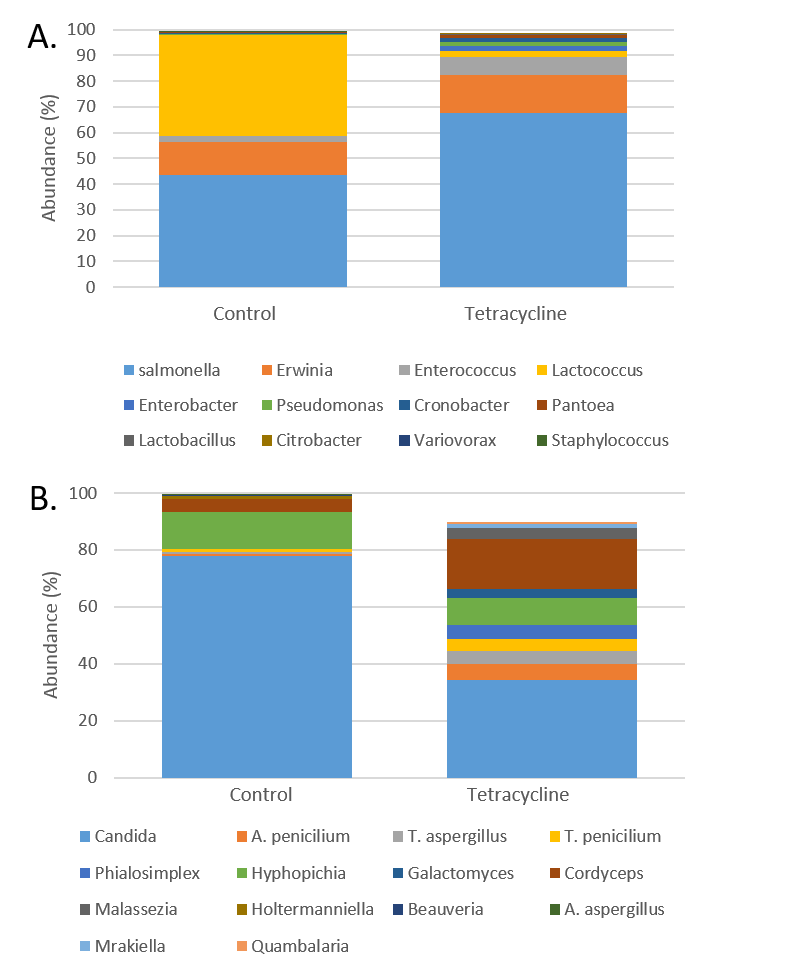

Supplement: S1 Fig — Relative abundance of the ten most abundant A) prokaryotic, and B) eukaryotic genera in relation to treatment at 48 h after treatment with Tetracycline (pooled data from beetles receiving either 5 mg mL-1 or 10 mg mL-1). Data represent the average relative abundance per treatment (control: n = 5, tetracycline: n = 10). (TIF) [file pone.0227561.s001.tif]

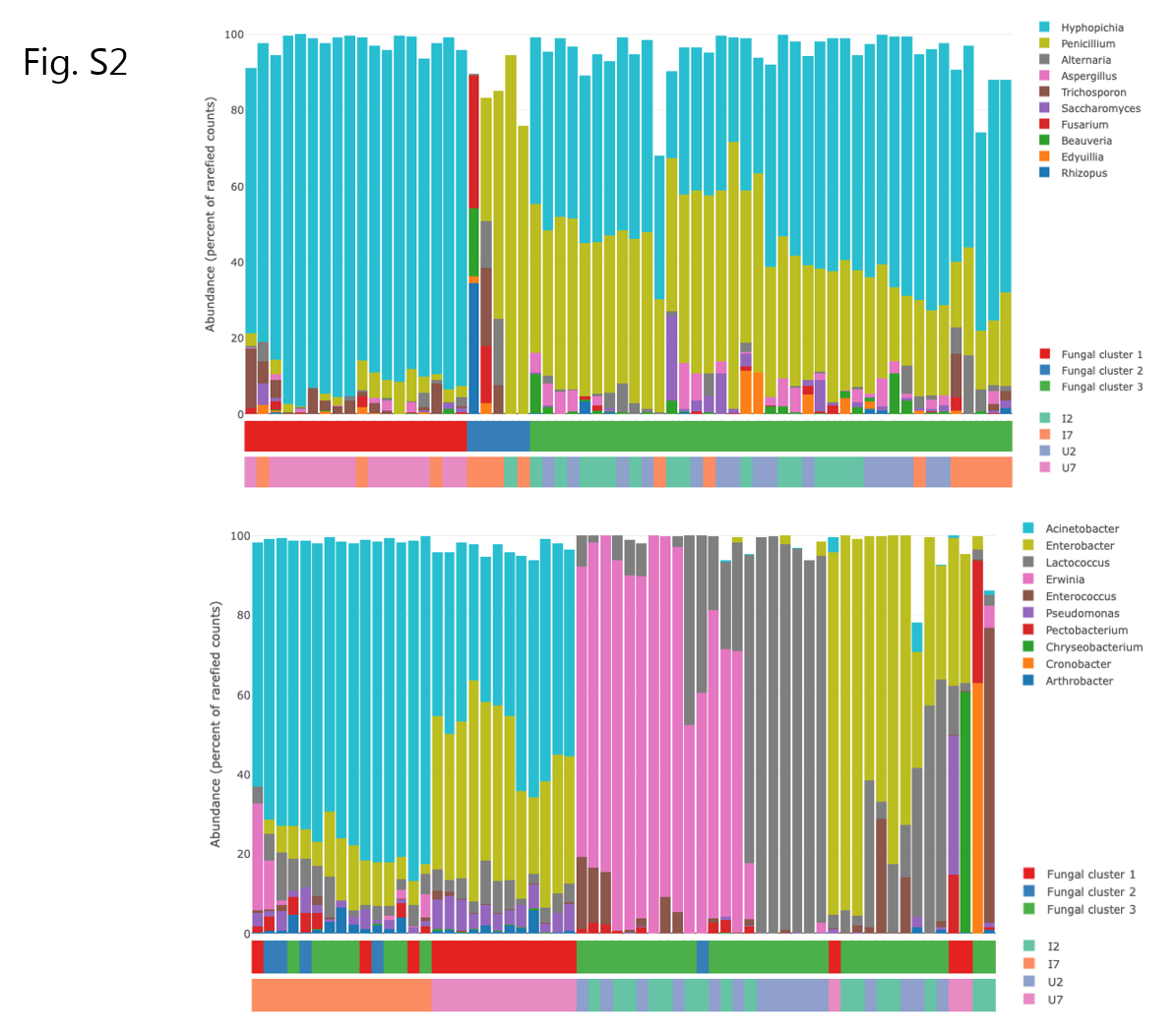

Supplement: S2 Fig — Barplots of the top 10 genera found in the mycobiome (A) and bacteriome (B) in the 62 samples which had data on both bacterial and fungal composition. Samples are ordered according to a hierarchical clustering of Bray-Curtis dissimilarities between samples, and three overall clusters of the mycobiome have been defined based on this clustering. These three clusters can largely be described as a Hyphopichia dominated cluster (cluster 1), a diverse cluster with no Hyphopichia at all (cluster 2), and a cluster where Pennicillium dominates along with Hyphopichia (cluster 3). Tiles below the barplots are color coded to show fungal cluster as well as time (day 2 or 7), and infection status (uninfected = U or infected = I) of samples in order to highlight concordances between bacteriome, mycobiome, and infection status. (TIF) [file pone.0227561.s002.tif]
